# *Rickettsia sibirica mongolitimonae* Infections in Spain and Case Review of the Literature

**DOI:** 10.3201/eid3101.240151

**Published:** 2025-01

**Authors:** Sonia Santibáñez, José Manuel Ramos-Rincón, Paula Santibáñez, Cristina Cervera-Acedo, Isabel Sanjoaquín, Encarnación Ramírez de Arellano, Sara Guillén, María del Carmen Lozano, Marta Llorente, Mario Puerta-Peña, Elena Aura Bularca, Alejandro González-Praetorius, Isabel Escribano, Lorenzo Sánchez, Valvanera Ibarra, Jorge Alba, Ana M. Palomar, Antonio Beltrán, Aránzazu Portillo, José A. Oteo

**Affiliations:** San Pedro University Hospital-Center for Biomedical Research of La Rioja, Logroño, Spain (S. Santibáñez, P. Santibáñez, C. Cervera-Acedo, V. Ibarra, J. Alba, A.M. Palomar, A. Portillo, J.A. Oteo); Dr. Balmis General University Hospital and Institute for Health and Biomedical Research, Alicante, Spain (J.M. Ramos-Rincón); Dr. Balmis General University Hospital, Alicante (I. Escribano); Lozano Blesa University Clinical Hospital, Zaragoza, Spain (I. Sanjoaquín, E.A. Bularca, A. Beltrán); Virgen Macarena University Hospital, Seville, Spain (E. Ramírez de Arellano); Getafe’s University Hospital and Centro de Investigación Biomédica en Red Enfermedades Infecciosas Instituto de Salud Carlos III, Madrid, Spain (S. Guillén); Virgen del Rocio University Hospital, Seville (M.C. Lozano); University Hospital of the Southeast, Madrid (M. Llorente); 12 de Octubre University Hospital, Madrid (M. Puerta-Peña); University General Hospital Guadalajara, Guadalajara, Spain (A. González-Praetorius, L. Sánchez)

**Keywords:** *Rickettsia sibirica mongolitimonae*, rickettsiosis, spotted fever, lymphangitis, eschar, rash, tick bite, worldwide, bacteria, *Rickettsia*, vector-borne infections, zoonoses, Europe, Spain

## Abstract

*Rickettsia sibirica mongolitimonae* is an emerging cause of tickborne rickettsiosis. Since the bacterium was first documented as a human pathogen in 1996, a total of 69 patients with this infection have been reported in the literature. Because of the rising rate of *R. sibirica mongolitimonae* infection cases, we evaluated the epidemiologic and clinical features of 29 patients who had *R. sibirica mongolitimonae* infections confirmed during 2007–2024 at the Center for Rickettsiosis and Arthropod-Borne Diseases, the reference laboratory of San Pedro University Hospital–Center for Biomedical Research of La Rioja, Logroño, Spain. We also reviewed all cases published in the literature during 1996–2024, evaluating features of 94 cases of *R. sibirica mongolitimonae* infection (89 in Europe, 4 in Africa, and 1 in Asia). Clinicians should consider *R. sibirica mongolitimonae* as a potential causative agent of rickettsiosis, and doxycycline should be administered promptly to avoid clinical complications.

The bacterium *Rickettsia sibirica mongolitimonae* (formerly *R. mongolotimonae*) has become an emerging cause of tickborne rickettsiosis since the 1990s. *R. sibirica mongolitimonae* was first documented as a human pathogen in France in 1996 in a woman who manifested a febrile rash and a single inoculation eschar on the groin; a rope-like lymphangitis also developed in the patient from the eschar to the draining lymph node (*1*). Four years later, *R. sibirica mongolitimonae* infection was diagnosed in a second patient, also in France. That patient manifested an inoculation eschar on the leg, fever, and lymphangitis that expanded from the eschar to an enlarged and painful lymph node in the groin (*2*). The first case reported outside of Europe occurred in South Africa in 2004; a man manifested an inoculation eschar on a toe, fever, headache, and lymphangitis expanding from the eschar to an enlarged inguinal lymph node (*3*). The first case series of infections, published in 2005, reported 7 new case-patients in France, 1 of whom was a traveler returning from southern Algeria (*4*). Clinical symptoms in those patients were fever, eschar, rash, and lymphangitis, and because of the lymphangitis symptom, it was named lymphangitis-associated rickettsiosis (*4*). Since 2005, most cases have been reported in the Mediterranean area, including France, Greece, Portugal, Spain, Turkey, and North Macedonia (*5*–*10*), and in other geographic areas, such as Africa and Asia (*11*,*12*). The clinical spectrum of infections has broadened; the bacterium has been shown to cause retinal vasculitis, septic shock, myopericarditis, and encephalitis (*13*–*16*). Since 2014, *R. sibirica mongolitimonae* has also been implicated as an etiologic agent of scalp eschar and neck lymphadenopathy after tick bite syndrome (*17*).

Few case series have been published worldwide (*4*,*8*,*18*); a total of 69 patients with *R. sibirica mongolitimonae* infections have been reported in case series or as isolated cases. Of those 69 patients, >30% (n = 22) were reported in Spain, the first of which was described at the Center for Rickettsiosis and Arthropod-Borne Diseases (CRETAV) in La Rioja, Spain (*7*). All of those cases were autochtonous. CRETAV, located at the Center for Biomedical Research of La Rioja, is a specialized laboratory and a center of excellence within the Network of Biologic Alert Laboratories that supports research on special pathogens, including those transmitted by ticks and other arthropods. CRETAV is also the reference laboratory for San Pedro University Hospital in La Rioja, receiving samples from other health services throughout the country. Thus, from June 2007 (when the first case was described) (*7*) through May 2024, *R. sibirica mongolitimonae* infections were confirmed at CRETAV in 29 of 365 patients who had tickborne rickettsioses. Diagnoses in the remaining 336 patients were *Dermacentor*-borne necrosis erythema lymphadenopathy (n = 187), boutonneuse fever (n = 130), African tick bite fever (n = 12, imported cases), and 5 other *Rickettsia* infections: *R*. *helvetica* (n = 2), *R*. *massiliae* (n = 2, 1 imported), *R*. *monacensis* (n = 1), *R*. *aeschlimannii* (n = 1), and *R*. *parkeri* (n = 1, imported case). Aware of the rising rate of patients with *R. sibirica mongolitimonae* infections diagnosed at CRETAV since 2020, and because only 4 cases from this 29-case series had been previously reported (*7*,*14*,*15*,*19*), we described the epidemiologic and clinical features of patients with confirmed *R. sibirica mongolitimonae* infections processed at CRETAV. We also reviewed all published cases because a comprehensive literature review was lacking. We obtained study approval from the regional ethics committee (Comité Ético de Investigación Clínica-Consejería de Sanidad de La Rioja; approval no. CEICLAR PI-37) and informed consent from all patients in this study. All procedures were in accordance with the ethical standards of the research committee and with the 1964 Helsinki declaration and its later amendments.

## Patients and Methods

### Infections Diagnosed at CRETAV

Patients were asked about medical antecedents during their clinical interview, and variables, including epidemiologic data, were written down in their medical chart. In those cases in which relevant information was not recorded, patients were called later and asked for those data. For children, information was confirmed by their parents. 

We defined a diagnosis of *R. sibirica mongolitimonae* infection on the basis of clinical suspicion (fever with or without rash, with or without eschar, and with or without lymphangitis) and positive PCR and sequencing results in patients with a history of tick bite or tick exposure. During June 2007–May 2024, cases were confirmed at CRETAV by using EDTA-blood samples, eschar biopsies, eschar swab samples, and tick samples from patients who were investigated at CRETAV because of clinical or epidemiologic suspicion of rickettsiosis. CRETAV diagnosed infections by using PCR of *ompA* and *ompB* genes corresponding to *R. sibirica mongolitimonae*. We subsequently reviewed clinical data of patients who had positive *ompA* and *ompB* PCR results for *R. sibirica mongolitimonae*.

Using molecular methods as described (*20*), we had previously extracted DNA from clinical samples from the Zoonosis Collection registered in the National Registry of Biobanks from Carlos III Health Institute (reference no. C.0006409), located at CRETAV–Centre of Biomedical Research of La Rioja. Whenever possible, we tested both acute phase and convalescent serum samples (collected 4–12 weeks apart), or only acute serum samples if the second sample was not available, by using immunofluorescence assays (IFA) to detect cross-reacting *R. conorii* IgG (CRETAV in-house assay or commercial assay [Vircell Microbiologists]). 

### Literature Review

We performed a systematic review of the literature by searching PubMed using the search terms “sibirica” or “mongolitimonae” or “mongolotimonae” and “*Rickettsia*” and a date range of January 1996–May 2024. We excluded nonhuman studies. We included human case reports and case series only if *R. sibirica mongolitimonae* infections were confirmed by either PCR and sequencing, except for 1 case, which was confirmed by indirect IFA and showed *R. sibirica mongolitimonae* IgG seroconversion. 

## Results

### *R. sibirica mongolitimonae* Infection Cases Diagnosed at CRETAV

**Figure 1:**
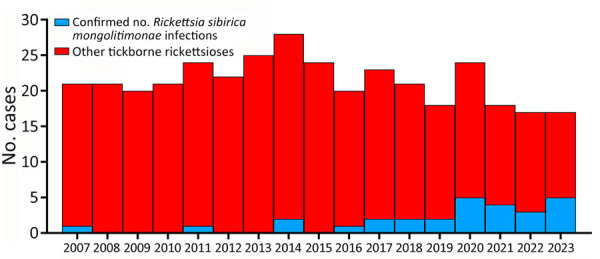
Number of confirmed rickettsioses cases in study of *Rickettsia sibirica mongolitimonae* infections in Spain. Numbers of patients with a confirmed case of *R. sibirica mongolitimone* infection and total numbers of other tickborne rickettsioses are indicated for each year during 2007–2023. Cases were diagnosed at the Center for Rickettsiosis and Arthropod-Borne Diseases, La Rioja, Spain.

**Figure 2:**
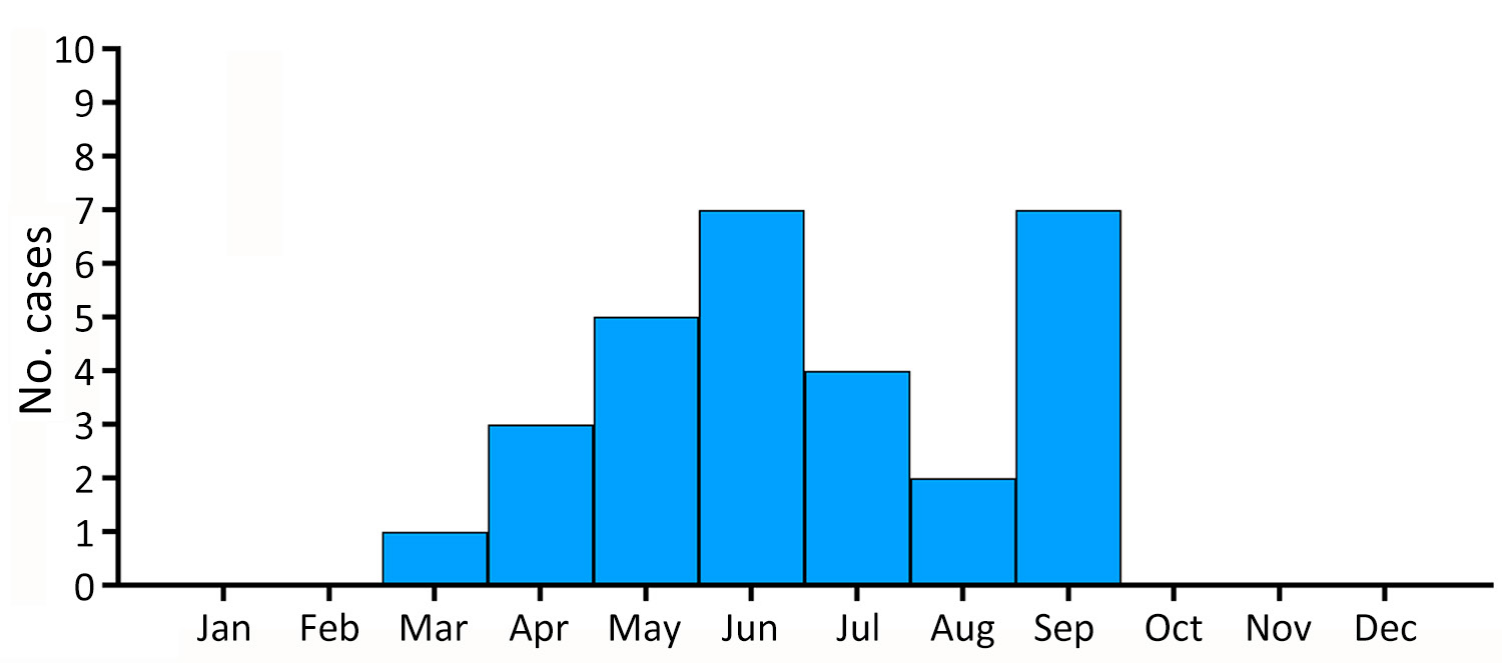
Monthly prevalence of *Rickettsia sibirica mongolitimonae* infections in Spain during 2007–May 2024. Patients sought medical care for *R. sibirica mongolitimonae* infections during March–September of each year.

Tickborne rickettsiosis caused by *R. sibirica mongolitimonae* was confirmed in 29 (7.9%) of 365 patients during 2007–2024. The number of *R. sibirica mongolitimonae* infections compared with the total number of tickborne *Rickettsia* spp. infections increased from 12.5% to 41.7% during 2020–2023 (Figure 1). Twenty-three (79.3%) of 29 patients were men, 6 (20.7%) women. The mean age was 58 (range 5–82) years; the median age was 67 years. Three (10.3%) patients were <15 years of age. All patients sought medical care during March–September of each year: 1 in March, 3 in April, 5 in May, 7 in June, 4 in July, 2 in August, and 7 in September (Figure 2). 

Only 6 (20.7%) patients remembered a tick bite, 1 had an attached tick, and 11 recalled tick exposure from hunting, gardening, living in a rural environment, or contact with dogs that had ticks. Patients resided in different regions in Spain: Aragón (n = 8), La Rioja (n = 8), Comunidad Valenciana (n = 4), Andalucía (n = 3), Madrid (n = 3), Vizcaya (n = 2) and Castilla-La Mancha (n = 1). The place of residence was within the same geographic region of the tick bite or exposure for all patients, even for those who did not recall activities associated with tick contact (n = 11) but denied recent travel outside of their residential region.

**Figure 3:**
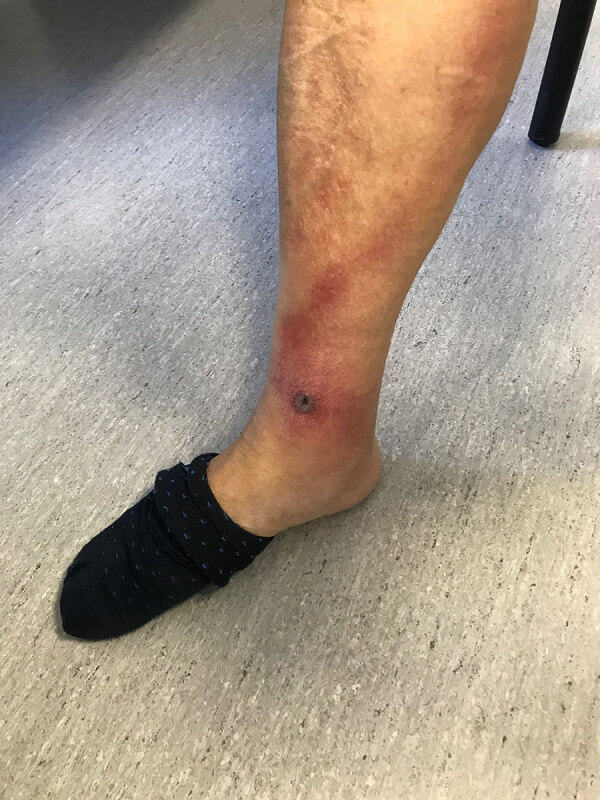
Eschar from *Rickettsia sibirica mongolitimonae* infection located on lower limb of patient in Spain.

**Figure 4:**
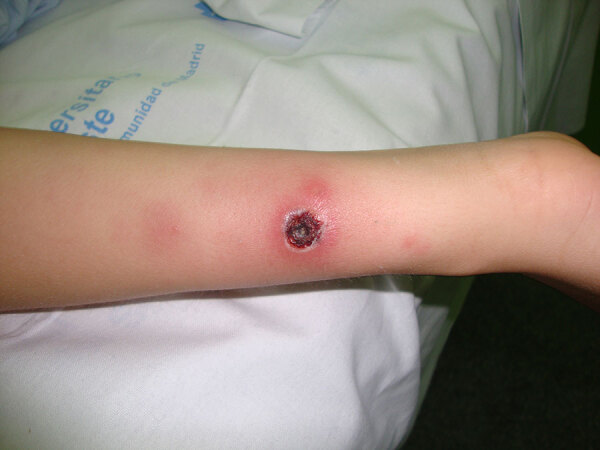
Eschar from *Rickettsia sibirica mongolitimonae* infection located on upper limb of a pediatric patient in Spain.

**Figure 5:**
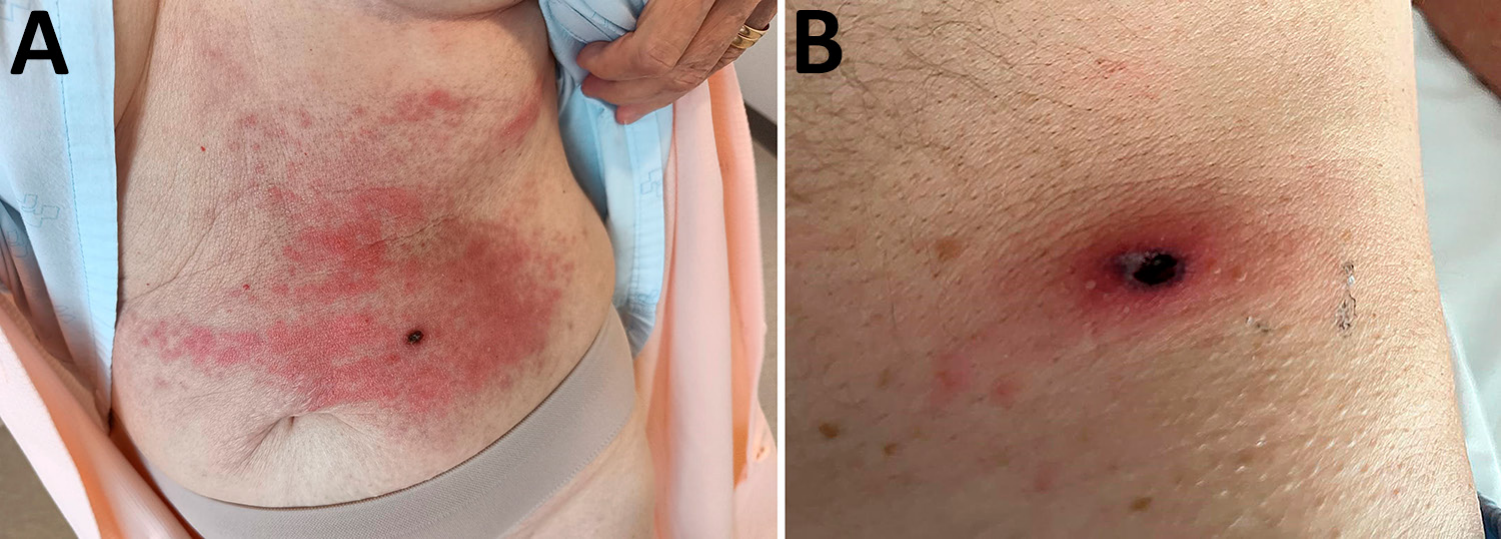
Eschars from *Rickettsia sibirica mongolitimonae* infections of 2 patients in Spain, located on the abdomen of 1 patient (A) and on the iliac fossa of another patient (B).

Symptoms at disease onset included fever (dysthermia, fever detected by thermometer) for all patients (Appendix Table 1). Inoculation eschars manifested in 27/29 (93.1%) patients as single (n = 20 [69.0%]) or multiple (n = 7 [24.1%]) eschars. They were located on lower limbs (n = 9 patients) (Figure 3), upper limbs (n = 7) (Figure 4), head (n = 3), hips (n = 3), buttocks (n = 3), groin (n = 2), abdomen (n = 2) (Figure 5, panel A), iliac fossa (n = 1) (Figure 5, panel B) and scrotum (n = 1) (*19*). A rope-like lymphangitis from the eschar to the draining lymph node was detected in 10/29 (34.5%) patients (Figure 3), and a generalized maculopapular rash was observed in 14/29 (48.3%) patients. One patient experienced septic shock and myopericarditis developed in another patient who had no remarkable medical history (*14*,*15*).

Hematologic and biochemical parameters were missing for several patients. When available, laboratory investigations showed leukopenia, thrombocytopenia, and raised lactate dehydrogenase, C-reactive protein, and liver enzymes (alanine aminotransferase, aspartate aminotransferase, and gamma glutamyl transaminase) as main findings.

We administered doxycycline (100 mg 2×/d for 10–14 days) to 26 patients, whereas 3 patients (2 children <15 years of age and 1 pregnant woman) received azithromycin (10 mg/kg 1×/d for 5 days for the children and 500 mg/kg 1×/d for 5 days for the adult). We observed improvement of signs and symptoms in all cases. We added supportive therapy with fluids and inotropic agents and intravenous meropenem (1 g every 8 hours) and vancomycin (1 g every 12 hours) for the patient who had septic shock.

For microbiologic tests, 1 clinical sample was available for 22/29 patients: EDTA-blood sample (n = 4), eschar biopsy (n = 5), and eschar swab sample (n = 12) (Appendix Table 1). For 1 patient, the only available clinical sample was the attached tick, which was identified as a *Rhipicephalus pusillus*. The remaining 7 patients had 2 different clinical samples available: eschar biopsy and eschar swab samples (n = 4), eschar swab and EDTA-blood samples (n = 2), and eschar biopsy and EDTA-blood sample (n = 1).

The *ompA* and *ompB* gene sequences obtained from 7 blood samples, 7 eschars, 18 eschar swab samples, and the *R. pusillus* tick showed the 100% similarity to those genes from *R. sibirica mongolitimonae* (GenBank accession no. MF379309 for *ompA* and JQ782657 for *ompB*). Acute- and convalescent-phase serum samples were obtained from 7 of 29 patients (Appendix Table 1). For 6 of those samples, we observed seroconversion (n = 5) or a 4-fold increase in titer (n = 1). We did not detect IgG against spotted fever group (SFG) *Rickettsia* in either serum sample for the remaining patient. For 6 patients, only acute serum samples were available; 2 of those showed IgG titers against *Rickettsia* and 4 did not react. No serum specimens were available for the remaining patients (Appendix Table 1). 

In July 2023, *ompA* and *ompB* genes from *R. sibirica mongolitimonae* (100% identity) were amplified by PCR in 1 *R. pusillus* tick attached to a 70-year-old patient. Five days after removing the tick, he had a high fever (39°C); an eschar at the armpit appeared 1 day later. No lymphangitis, lymphadenopathies, or rash were observed. No EDTA-blood, eschar biopsy, or eschar swab samples were available from that patient. He was treated with doxycycline and recovered.

### Published *R. sibirica mongolitimonae* Infection Cases, Including This Study

We found, reviewed, and extracted data from the full text of 26 selected papers from PubMed. Ninety-four cases of *R. sibirica mongolitimonae* infection were reported worldwide during January 1996–May 2024, including the 29 cases from this study; 89 of those cases were reported in Europe: Spain (n = 47), France (n = 36), Greece (n = 2), Portugal (n = 2), Turkey (n = 1), and Macedonia (n = 1) (Appendix Table 2). Only 4 cases have been published in Africa, including South Africa (unique case occurred in southern hemisphere), Algeria, Egypt and Cameroon, and 1 in Asia (Sri Lanka) (Appendix Table 2).

**Figure 6:**
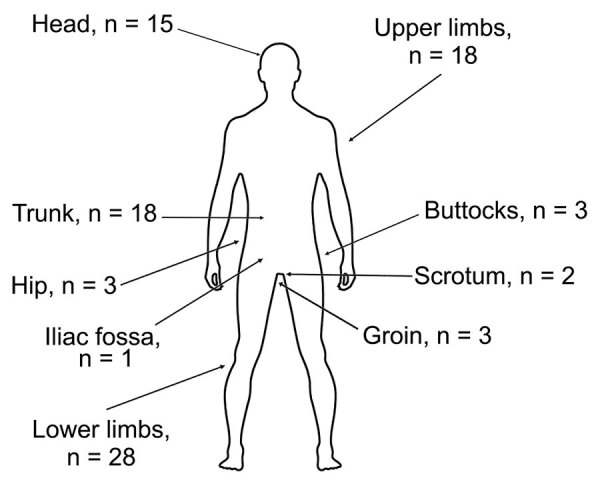
Numbers and body locations of tick bites and inoculation eschars in patients with *Rickettsia sibirica mongolitimonae* infections during 2007–2024, Spain, and from published literature. Patients manifested single or multiple eschars. Cases were diagnosed at the Center for Rickettsiosis and Arthropod-Borne Diseases, La Rioja, Spain and/or identified in PubMed.

Of the total number of patients described, 67.0% were men and 31.9% women. The mean age was 49.8 (range 4–82) years; the median age was 55.5 years. Most (n = 77) infection cases occurred during April–July and in September of each year. Only ≈33% of patients remembered a tick bite. Four patients kept the ticks (1 tick/person), which were identified as female *Hyalomma marginatum*, *H. anatolicum excavatum*, *Hyalomma* sp., and *R. pusillus* ticks (*5*,*9*,*10*). The symptoms at disease onset (always after tick removal) included fever for all patients; ≈95% of patients manifested inoculation eschars, and 14 patients had multiple eschars. Eschars were located on lower limbs (n = 28), upper limbs (n = 18), trunk (n = 18), head (n = 15), hip (n = 4) and iliac fossa (n = 1), groin (n = 3), scrotum (n = 2), and buttocks (n = 3) (Figure 6). A rope-like lymphangitis from the eschar to the draining lymph node was noted in 33 (35.1%) of 94 published cases (Figure 3); a generalized maculopapular rash was observed in 66.7% of cases.

In published cases, *R. sibirica mongolitimonae* genes were amplified by PCR in 43 eschar biopsy specimens, 38 eschar swab samples, 12 blood samples, and 3 ticks (*H. anatolicum excavatum, H. marginatum*, and *R. pusillus*) removed from infected patients. Ten patients had >1 PCR-positive clinical sample: eschar biopsy and eschar swab sample (n = 5), eschar biopsy and blood sample (n = 2), eschar swab and blood samples (n = 2), and blood sample and a tick specimen (n = 1). Most (91.9%) patients were treated with doxycycline for 7–14 days, and all patients recovered without sequelae after antimicrobial drug treatment.

## Discussion

We describe the epidemiologic and clinical characteristics of *R. sibirica mongolitimonae* infections in 29 patients who had their infection confirmed at CRETAV in Spain during 2007–2024, and we reviewed all available cases published in the literature during January 1996–May 2024. *R. sibirica mongolitimonae* has been considered a rare pathogen. Nevertheless, this bacterium has been the causative agent of >94 rickettsiosis cases since 1996, including the case series described in this study, mostly in the Mediterranean area. This infection typically manifests with high fever, myalgia and headache, single or multiple inoculation eschars with a nondefined inflammatory halo, nonpruritic maculopapular rash involving palms and soles, and enlarged draining lymph nodes. Lymphangitis is a typical sign of this infection and occurs in ≈40% of infected patients. Lymphangitis might occur in other rickettsioses, such as those caused by *R. heilongjiangensis* and *R. africae*, although in lesser proportions. Thus, in Europe, the presence of lymphangitis should suggest *R. sibirica mongolitimonae* infection (*21*). In this study, 10 (34.5%) of the 29 patients with *R. sibirica mongolitimonae* infections diagnosed at CRETAV had lymphangitis, a slightly higher percentage than that shown in a previous report from France in 2016 (*18*); a rope-like lymphangitis from the eschar to the draining lymph node was observed in 33/94 (35.1%) cases published worldwide. Fever was present in 100% of published cases; a generalized maculopapular rash was observed in 14 (48.3%) of 29 patients with infections confirmed at CRETAV, whereas 67.0% of patients described in published cases had the rash. Inoculation eschars were noted in 27 (93.1%) of 29 patients with infections confirmed at CRETAV and in 89 (94.7%) patients from published cases. Multiple inoculation eschars developed in 13 (13.8%) patients in published cases; a higher (24.1%) percentage was found in CRETAV cases. The presence of multiple inoculation eschars is common in patients with African tick bite fever (*22*).

*R. sibirica mongolitimonae* infection frequently causes a mild, nonlethal disease, but some complications have been described, such as septic shock, disseminated intravascular coagulation, neurologic disorders, acute renal failure, and myopericarditis (*13*–*16*,*23*). In 2020, a case of *R. sibirica mongolitimonae* infection with associated encephalitis was reported (*16*).

All outcomes, including for severe cases, were successful after antimicrobial drug administration. Doxycycline is the drug of choice for treating rickettsioses, even for children <8 years of age (*24*). Macrolides, such as azithromycin, are effective against rickettsial diseases and can be safely used during pregnancy. The early start of empirical treatment reduces severity and duration of symptoms (*25*). For published cases, oral doxycycline (100 mg/12 hours for 7–15 days) was administered to all patients, except 4 who received azithromycin (1 man, 1 pregnant woman, and 2 children 4 and 6 years of age). One patient received pristinamycin for 7 days.

In accordance with previously published reports, we found that *R. sibirica mongolitimonae* infections were seasonal and most cases occurred during the spring and summer (April–July) and in September. The infections affected men more frequently than women. In our case series, 23 (79.3%) patients were men and 6 (20.7%) were women, compared with 67.0% men and 31.9% women in published cases. The mean age was 58 years in the case series and 50 years in the published cases.

*R. sibirica mongolitimonae* infections are likely underdiagnosed or misdiagnosed as another rickettsiosis because diagnosis is mainly made according to serologic testing, which includes IFAs. Serology is limited by cross-reactions with other *Rickettsia* spp., mostly among SFG *Ricketsia* spp. because they share antigenic characteristics. Using molecular tools, such as PCR and quantitative PCR–based methods, on skin biopsy (eschar) and eschar swab samples appear to be the best methods to detect and identify *R. sibirica mongolitimonae*. For molecular diagnosis, eschar swab samples are preferred over skin biopsies because the sampling procedure is noninvasive and highly effective (*26*–*28*). Culture as a diagnostic method is fastidious and performed only in reference laboratories. Nevertheless, 6 eschar biopsies were positive for *R. sibirica mongolitimonae* by culture methods in published cases (*1*,*2*,*4*,*6*).

*R. sibirica mongolitimonae* was initially isolated from *H.* *asiaticum* ticks in Inner Mongolia (*29*) and from *H. truncatum* ticks in Niger (*30*). In Europe, *R. sibirica mongolitimonae* was detected in *H. excavatum* ticks in Greece and Cyprus, in *H. marginatum* ticks in Spain (*31**,**32*), in *R. pusillus* ticks in Portugal, Spain and France (*6*,*31*,*33*), and in *R*. *bursa* ticks in Spain (*34*). In Turkey, it was also detected in *R. bursa*, *Haemaphysalis parva*, *H. excavatum*, and *H. marginatum* ticks (*35*).

In 2005, the presence of *R. sibirica mongolotimonae* was reported both in a patient and in a *H*. *anatolicum excavatum* tick removed from that patient in Greece (*5*). In 2016, in Turkey, *R. sibirica mongolitimonae* infection was diagnosed by PCR in a man who had been bitten by a *H. marginatum* tick (*9*). A case of *R. sibirica mongolitimonae* infection after a *Hyalomma* sp. tick bite has been recently reported in North Macedonia (*10*). No ticks were associated with the remaining published cases, and many patients did not even remember receiving a tick bite. Nevertheless, because *R. sibirica mongolitimonae* has been detected in *Rhipicephalus* spp. ticks collected from areas close to where infected patients lived (*6*,*33*), *Rhipicephalus* ticks are also suspected vectors in Europe.

In published cases, only 18 (19.1%) of 94 patients remembered a tick bite. Four patients kept the ticks, which were identified as *H. marginatum*, *H. anatolicum excavatum*, *Hyalomma* sp., and *R. pusillus*; *R. sibirica mongolitimonae* was amplified in 3 of those ticks. *R. sibirica mongolitimonae* has been most frequently associated with *Hyalomma* ticks, the confirmed vectors in Africa. However, adult *Hyalomma* spp. ticks are large, but most patients did not remember a tick bite. This finding suggests that either the *Hyalomma* spp. vector is at an immature stage, which is rare because few bites occur from *Hyalomma* larvae and nymphs, or the vector could also be *R. pusillus*, which is a small tick found in rabbits. Data from case number 93 (Appendix Table 2), obtained from this case series, strengthens the potential role of *R. pusillus* ticks as *R. sibirica mongolitimonae* vectors. Under a One Health perspective, excessive reproduction of rabbits in urban/peri-urban areas of cities might cause human cases of *R. sibirica mongolitimonae* infection.

In Europe, *R. sibirica mongolitimonae* was confirmed as a human pathogen in 1996, and 94 cases have been reported during 1996–2024. During 1996–2012, only 25 cases were published, whereas during 2013–2024, the number of published cases reached 70. This upward trend of reports might be partly caused by the use of new tools to investigate tick-transmitted agents. In addition, warming weather and the overgrowth of certain wildlife species, among other factors, are involved in the increase in tick threats. Thus, the expansion of wild boar and rabbit populations might favor an increase in adult tick species responsible for *R. sibirica mongolitimonae* transmission. *Hyalomma* spp. ticks are characterized by their aggressive host-seeking behavior, unlike other tick species that use a passive ambush strategy as they wait in vegetation. Although *Hyalomma* ticks are not particularly anthropophilic, a progressive increase in their population has been reported in Spain and other areas in Europe, probably related to factors previously mentioned.

A strength of this study lies in the large number of clinical cases in Spain that were evaluated, accompanied by a literature review. However, it is possible that not all case data in Spain were collected because, despite the bibliographic search, *R. sibirica mongolitimonae* infections diagnosed in patients at other centers might not have been reported.

In conclusion, when rickettsiosis is clinically suspected, clinicians should be aware that empiric therapy should not wait for microbiologic confirmation. Doxycycline must be administered promptly, even in children, to avoid clinical complications. The rapid identification of *Rickettsia* spp. by using molecular techniques to analyze swab samples from inoculation eschars should be systematized. Because of the broad clinical spectrum of *R. sibirica mongolitimonae* infections, this emerging rickettsiosis is likely underdiagnosed or misdiagnosed as another SFG rickettsiosis. Clinicians should consider *R. sibirica mongolitimonae* as a potential causative agent in patients who have fever and an eschar or rash with or without lymphangitis and should consider the epidemiologic context.

Appendix Additional information for *Rickettsia sibirica mongolitimonae* infections in Spain and case review of the literature.

## References

[1] Raoult D, Brouqui P, Roux V. A new spotted-fever-group rickettsiosis. Lancet. 1996;348:412. 10.1016/S0140-6736(05)65037-4 8709763

[2] Fournier PE, Tissot-Dupont H, Gallais H, Raoult DR. *Rickettsia mongolotimonae*: a rare pathogen in France. Emerg Infect Dis. 2000;6:290–2. 10.3201/eid0603.000309 10827119 PMC2640873

[3] Pretorius AM, Birtles RJ. *Rickettsia mongolotimonae* infection in South Africa. Emerg Infect Dis. 2004;10:125–6. 10.3201/eid1001.020662 15078607 PMC3031104

[4] Fournier PE, Gouriet F, Brouqui P, Lucht F, Raoult D. Lymphangitis-associated rickettsiosis, a new rickettsiosis caused by *Rickettsia sibirica mongolotimonae*: seven new cases and review of the literature. Clin Infect Dis. 2005;40:1435–44. 10.1086/429625 15844066

[5] Psaroulaki A, Germanakis A, Gikas A, Scoulica E, Tselentis Y. Simultaneous detection of “*Rickettsia mongolotimonae*” in a patient and in a tick in Greece. J Clin Microbiol. 2005;43:3558–9. 10.1128/JCM.43.7.3558-3559.2005 16000506 PMC1169122

[6] de Sousa R, Barata C, Vitorino L, Santos-Silva M, Carrapato C, Torgal J, et al. *Rickettsia sibirica* isolation from a patient and detection in ticks, Portugal. Emerg Infect Dis. 2006;12:1103–8. 10.3201/eid1207.051494 16836827 PMC3291052

[7] Aguirrebengoa K, Portillo A, Santibáñez S, Marín JJ, Montejo M, Oteo JA. Human *Rickettsia sibirica mongolitimonae* infection, Spain. Emerg Infect Dis. 2008;14:528–9. 10.3201/eid1403.070987 18494099 PMC2570842

[8] Ramos JM, Jado I, Padilla S, Masiá M, Anda P, Gutiérrez F. Human infection with *Rickettsia sibirica mongolitimonae*, Spain, 2007-2011. Emerg Infect Dis. 2013;19:267–9. 10.3201/eid1902.111706 23343524 PMC3559030

[9] Kuscu F, Orkun O, Ulu A, Kurtaran B, Komur S, Inal AS, et al. *Rickettsia sibirica mongolitimonae* Infection, Turkey, 2016. Emerg Infect Dis. 2017;23:1214–6. 10.3201/eid2307.170188 28628458 PMC5512508

[10] Jakimovski D, Mateska S, Simin V, Bogdan I, Mijatović D, Estrada-Peña A, et al. Mediterranean spotted fever-like illness caused by *Rickettsia sibirica mongolitimonae*, North Macedonia, June 2022. Euro Surveill. 2022;27:2200735. 10.2807/1560-7917.ES.2022.27.42.2200735 36268740 PMC9585876

[11] Cordier C, Tattevin P, Leyer C, Cailleaux M, Raoult D, Angelakis E. *Rickettsia sibirica mongolitimonae* infection, Sri Lanka. J Infect Dev Ctries. 2017;11:668–71. 10.3855/jidc.8743 31085830

[12] Nouchi A, Monsel G, Jaspard M, Jannic A, Angelakis E, Caumes E. *Rickettsia sibirica mongolitimonae* infection in a woman travelling from Cameroon: a case report and review of the literature. J Travel Med. 2018;25:25. 10.1093/jtm/tax074 29394384

[13] Caron J, Rolain JM, Mura F, Guillot B, Raoult D, Bessis D. *Rickettsia sibirica* subsp. *mongolitimonae* infection and retinal vasculitis. Emerg Infect Dis. 2008;14:683–4. 10.3201/eid1404.070859 18394301 PMC2570939

[14] Ibarra V, Portillo A, Palomar AM, Sanz MM, Metola L, Blanco JR, et al. Septic shock in a patient infected with *Rickettsia sibirica mongolitimonae*, Spain. Clin Microbiol Infect. 2012;18:E283–5. 10.1111/j.1469-0691.2012.03887.x 22548679

[15] Revilla-Martí P, Cecilio-Irazola Á, Gayán-Ordás J, Sanjoaquín-Conde I, Linares-Vicente JA, Oteo JA. Acute myopericarditis associated with tickborne *Rickettsia sibirica mongolitimonae.* Emerg Infect Dis. 2017;23:2091–3. 10.3201/eid2312.170293 29148392 PMC5708254

[16] Loarte MDC, Melenotte C, Cassir N, Cammilleri S, Dory-Lautrec P, Raoult D, et al. *Rickettsia mongolitimonae* encephalitis, southern France, 2018. Emerg Infect Dis. 2020;26:362–4. 10.3201/eid2602.181667 31961319 PMC6986838

[17] Dubourg G, Socolovschi C, Del Giudice P, Fournier PE, Raoult D. Scalp eschar and neck lymphadenopathy after tick bite: an emerging syndrome with multiple causes. Eur J Clin Microbiol Infect Dis. 2014;33:1449–56. 10.1007/s10096-014-2090-2 24682865

[18] Angelakis E, Richet H, Raoult D. *Rickettsia sibirica mongolitimonae* Infection, France, 2010-2014. Emerg Infect Dis. 2016;22:880–2. 10.3201/eid2205.141989 27088367 PMC4861502

[19] Salazar Alarcón E, Guillén-Martín S, Callejas-Caballero I, Valero-Arenas A. Clinical case report: Not all rickettsiosis are mediterranean spotted fever. Enferm Infecc Microbiol Clin (Engl Ed). 2022;40:44–5. 10.1016/j.eimce.2021.10.004 34732342

[20] Santibáñez S, Portillo A, Ibarra V, Santibáñez P, Metola L, García-García C, et al. Epidemiological, clinical, and microbiological characteristics in a large series of patients affected by *Dermacentor*-borne-necrosis-erythema-lymphadenopathy from a unique centre from Spain. Pathogens. 2022;11:528. 10.3390/pathogens11050528 35631049 PMC9146834

[21] Faccini-Martínez ÁA, García-Álvarez L, Hidalgo M, Oteo JA. Syndromic classification of rickettsioses: an approach for clinical practice. Int J Infect Dis. 2014;28:126–39. 10.1016/j.ijid.2014.05.025 25242696

[22] Althaus F, Greub G, Raoult D, Genton B. African tick-bite fever: a new entity in the differential diagnosis of multiple eschars in travelers. Description of five cases imported from South Africa to Switzerland. Int J Infect Dis. 2010;14(Suppl 3):e274–6. 10.1016/j.ijid.2009.11.021 20233665

[23] Gaillard E, Socolovschi C, Fourcade C, Lavigne JP, Raoult D, Sotto A. [A case of severe sepsis with disseminated intravascular coagulation during *Rickettsia sibirica mongolitimonae* infection] [in French]. Med Mal Infect. 2015;45:57–9. 10.1016/j.medmal.2014.10.005 25455075

[24] Todd SR, Dahlgren FS, Traeger MS, Beltrán-Aguilar ED, Marianos DW, Hamilton C, et al. No visible dental staining in children treated with doxycycline for suspected Rocky Mountain Spotted Fever. J Pediatr. 2015;166:1246–51. 10.1016/j.jpeds.2015.02.015 25794784

[25] Brouqui P, Bacellar F, Baranton G, Birtles RJ, Bjoërsdorff A, Blanco JR, et al.; ESCMID Study Group on Coxiella, Anaplasma, Rickettsia and Bartonella; European Network for Surveillance of Tick-Borne Diseases. Guidelines for the diagnosis of tick-borne bacterial diseases in Europe. Clin Microbiol Infect. 2004;10:1108–32. 10.1111/j.1469-0691.2004.01019.x 15606643

[26] Solary J, Socolovschi C, Aubry C, Brouqui P, Raoult D, Parola P. Detection of *Rickettsia sibirica mongolitimonae* by using cutaneous swab samples and quantitative PCR. Emerg Infect Dis. 2014;20:716–8. 10.3201/eid2004.130575 24655579 PMC3966388

[27] Wang JM, Hudson BJ, Watts MR, Karagiannis T, Fisher NJ, Anderson C, et al. Diagnosis of Queensland tick typhus and African tick bite fever by PCR of lesion swabs. Emerg Infect Dis. 2009;15:963–5. 10.3201/eid1506.080855 19523304 PMC2727311

[28] Bechah Y, Socolovschi C, Raoult D. Identification of rickettsial infections by using cutaneous swab specimens and PCR. Emerg Infect Dis. 2011;17:83–6. 10.3201/eid1701.100854 21192860 PMC3375762

[29] Yu X, Jin Y, Fan M, Xu G, Liu Q, Raoult D. Genotypic and antigenic identification of two new strains of spotted fever group rickettsiae isolated from China. J Clin Microbiol. 1993;31:83–8. 10.1128/jcm.31.1.83-88.1993 8093253 PMC262626

[30] Parola P, Inokuma H, Camicas JL, Brouqui P, Raoult D. Detection and identification of spotted fever group Rickettsiae and Ehrlichiae in African ticks. Emerg Infect Dis. 2001;7:1014–7. 10.3201/eid0706.010616 11747731 PMC2631901

[31] Fernández de Mera IG, Ruiz-Fons F, de la Fuente G, Mangold AJ, Gortázar C, de la Fuente J. Spotted fever group rickettsiae in questing ticks, central Spain. Emerg Infect Dis. 2013;19:1163–5. 10.3201/eid1907.130005 23763913 PMC3713984

[32] Palomar AM, Portillo A, Mazuelas D, Roncero L, Arizaga J, Crespo A, et al. Molecular analysis of Crimean-Congo hemorrhagic fever virus and *Rickettsia* in *Hyalomma marginatum* ticks removed from patients (Spain) and birds (Spain and Morocco), 2009-2015. Ticks Tick Borne Dis. 2016;7:983–7. 10.1016/j.ttbdis.2016.05.004 27215620

[33] Edouard S, Parola P, Socolovschi C, Davoust B, La Scola B, Raoult D. Clustered cases of *Rickettsia sibirica mongolitimonae* infection, France. Emerg Infect Dis. 2013;19:337–8. 10.3201/eid1902.120863 23460995 PMC3559049

[34] Toledo A, Olmeda AS, Escudero R, Jado I, Valcárcel F, Casado-Nistal MA, et al. Tick-borne zoonotic bacteria in ticks collected from central Spain. Am J Trop Med Hyg. 2009;81:67–74. 10.4269/ajtmh.2009.81.67 19556569

[35] Keskin A, Bursali A, Keskin A, Tekin S. Molecular detection of spotted fever group rickettsiae in ticks removed from humans in Turkey. Ticks Tick Borne Dis. 2016;7:951–3. 10.1016/j.ttbdis.2016.04.015 27131413

