# Supplementary material for: Rickettsia sibirica mongolitimonae Infections in Spain and Case Review of the Literature
Source: Emerg Infect Dis. 2025 Jan;31(1):18–26. doi: 10.3201/eid3101.240151 (PMC11682799; doi:10.3201/eid3101.240151)
Supplement: Appendix — Additional information for Rickettsia sibirica mongolitimonae infections in Spain and case review of the literature. [file 24-0151-Techapp-s1.pdf]

Article DOI: https://doi.org/10.3201/eid3101.240151
EID cannot ensure accessibility for supplementary materials supplied by authors. Readers who have difficulty accessing supplementary content should contact the authors for assistance.

Rickettsia sibirica mongolitimonae Infections in Spain and Case Review of the Literature

Appendix

Appendix Table 1. Detailed data about patients with *R. sibirica mongolitimonae* infections confirmed at the Center for Rickettsiosis and Arthropod-Borne Diseases (CRETAV) in Rioja, Spain, during 2007–2024

Case no. (ref)	Year	Bite/eschar location	No. eschars	Fever	Rash	Lymphangitis	Hospitalized	Available clinical samples	PCR+ sample	DNA amplicon size, bp*		IgG titers†, acute/convalescent
										ompA	*ompB*	
1 (*1*)	2007	Leg	None	Yes	No	No	No	Blood	Blood	486	382	Negative/negative
2 (*2*)	2011	Hip	1	Yes	Yes	No	Yes	Blood	Blood	486	381	Negative/1:512
3	2014	Forearm	1	Yes	Yes	Yes	Yes	Eschar swab	Eschar swab	503	359	No serum
4	2014	Groin	1	Yes	No	No	No	Eschar swab	Eschar swab	484	453	1:128/1:2,048
5 (*3*)	2016	Buttocks	1	Yes	No	No	Yes	Blood, eschar, eschar swab	Eschar, eschar swab	481	464	Negative/1:1,024
6	2017	Scalp	2	Yes	No	No	No	Blood, eschar swab	Blood, eschar swab	491	359	1:1,024/no serum
7	2017	Foot	1	Yes	No	Yes	Yes	Eschar swab	Eschar swab	491	382	Negative/no serum
8	2018	Forearm	1	Yes	No	No	No	Eschar swab	Eschar swab	515	464	No serum
9	2018	Shoulder	2	Yes	Yes	No	Yes	Eschar, eschar swab	Eschar, eschar swab	481	464	No serum
10	2019	Scapula/leg	2	Yes	No	No	No	Eschar swab	Eschar swab	484	453	No serum
11	2019	Leg	2	Yes	No	No	Yes	Eschar swab	Eschar swab	491	382	No serum
12	2020	Leg	2	Yes	Yes	Yes	Yes	Blood, eschar swab	Eschar swab	461	453	Negative/1:64
13 (*4*)	2020	Scrotum	1	Yes	Yes	No	Yes	Eschar	Eschar	491	464	No serum
14	2020	Scalp	1	Yes	Yes	Yes	No	Eschar swab	Eschar swab	486	452	Negative/no serum
15	2020	Ankle	1	Yes	Yes	Yes	Yes	Eschar swab	Eschar swab	491	464	No serum
16	2020	Thigh	1	Yes	Yes	No	No	Blood	Blood	486	382	Negative/no serum
17	2021	Thigh/hip	1	Yes	No	No	No	Eschar swab	Eschar swab	491	464	1:64/no serum
18	2021	Buttocks	1	Yes	Yes	Yes	Yes	Blood, eschar	Eschar	491	464	No serum
19	2021	Unknown	None	Yes	Yes	No	Yes	Blood	Blood	481	380	Negative/1:1,024
20	2021	Arm	2	Yes	Yes	No	Yes	Blood, eschar	Blood, eschar	481	464	No serum
21	2022	Hip	1	Yes	Yes	No	No	Blood, eschar, eschar swab	Eschar and eschar swab	491	382	No serum
22	2022	Face	1	Yes	No	No	No	Eschar swab	Eschar swab	491	382	No serum
23	2022	Arm	1	Yes	No	Yes	Yes	Eschar swab	Eschar swab	491	464	No serum
24	2023	Leg	1	Yes	No	Yes	Yes	Eschar swab	Eschar swab	491	464	No serum

Case no. (ref)	Year	Bite/eschar location	No. eschars	Fever	Rash	Lymphangitis	Hospitalized	Available clinical samples	PCR+ sample	DNA amplicon size, bp*		IgG titers†, acute/convalescent
										ompA	*ompB*	
25	2023	Abdomen/ groin	2	Yes	Yes	No	Yes	Eschar swab	Eschar swab	472	464	No serum
26	2023	Iliac fossa	1	Yes	No	No	Yes	Blood, eschar swab	Blood and eschar swab	481	464	Negative/1:1,024
27	2023	Abdomen	1	Yes	Yes	No	Yes	Eschar swab	Eschar swab	491	464	No serum
28	2023	Armpit	1	Yes	No	No	No	Tick	Tick	491	464	No serum
29	2024	Buttocks	1	Yes	No	No	No	Eschar swab	Eschar swab	491	459	Negative/no serum

*All PCR amplicons of *ompA* and *ompB* genes showed 100% identity with those of *Rickettsia sibirica mongolitimonae*. bp, base pairs; ref, reference; +, positive.
†IgG titers determined for acute and convalescent serum samples by using immunofluorescence assays.

Appendix Table 2. Epidemiologic, clinical, and microbiologic characteristics associated with patients infected with *Rickettsia sibirica mongolitimonae**

Case no.	Country	Year case occurred/publication	Patient age, y/sex	Tick bite reported	Other epidemiologic condition	Month†	Bite/eschar locations	No. eschars	Fever	Rash	Lymphangitis	PCR+ sample	Ref.
1	France	1996/1996	63/F	No	Gardening	March	Groin	1	Yes	Yes	Yes	Eschar	(*5*)
2	France	1998/2000	49/M	No	Gardener, living in rural area	May	Leg	1	Yes	No	Yes	Eschar	(*6*)
3	South Africa	2002/2004	34/M	No	Working in area with *Hyalomma truncatum*	September	Toe, foot	1	Yes	No	Yes	Eschar	(*7*)
4	France	2000–2004/2005	53/M	Yes	Gardening	May	Knee	1	Yes	Yes	No	Eschar	(*8*)
5	France	2000–2004/2005	40/F	Yes	Gardening	July	None	None	Yes	Yes	Yes	Serum	(*8*)
6	France	2000–2004/2005	59/M	Yes	Gardening	April	Back	1	Yes	Yes	No	Eschar	(*8*)
7	France	2000–2004/2005	21/F	No	Gardening	May	Heel	1	Yes	No	Yes	Serum	(*8*)
8	France	2000–2004/2005	70/M	No	Gardening, contact with birds	May	Forearm/ abdomen	2	Yes	Yes	No	Eschar	(*8*)
9	France	2000–2004/2005	55/M	No	Walk in Camargue National Park	June	Arm	1	Yes	Yes	Yes	Eschar	(*8*)
10	France, return from Algeria	2000–2004/2005	62/F	No	Travel to southern Algeria	April	Foot, hypochondrium	2	Yes	Yes	No	Eschar, serum	(*8*)
11	Crete	2002/2005	76/M	Yes, *H. anatolicum excavatum*	Collecting olives	December	Scrotum	None	Yes	Yes	No	Blood, tick	(*9*)
12	Portugal	2004/2006	73/F	No	None	August	Toe	1	Yes	Yes	No	Blood culture	(*10*)
13	France	2005/2008	20/F	No	Walking	June	Face	1	Yes	Yes	No	Eschar	(*11*)
14	Portugal	2006/2008	44/M	No	Vacation	August	Forearm	1	Yes	Yes	Yes	Eschar	(*12*)
15	Spain	2007/2008	41/M	Yes, tick removed	Topographer	June	Leg	None	Yes	No	No	Blood	(*1*)
16	Spain	2007/2013	67/F	No	Gardening	July	Scalp	1	Yes	Yes	No	Eschar	(*13*)
17	Spain	2009/2013	32/M	No	Working at golf courses	September	Thigh	1	Yes	Yes	Yes	Eschar	(*13*)
18	Spain	2010/2013	33/M	No	Walking in rural area	April	Leg	1	Yes	No	Yes	Eschar	(*13*)

Case no.	Country	Year case occurred/publication	Patient age, y/sex	Tick bite reported	Other epidemiologic condition	Month†	Bite/eschar locations	No. eschars	Fever	Rash	Lymphangitis	PCR+ sample	Ref.
19	Spain	2011/2013	42/F	No	Walking in rural area	March	Shoulder	1	Yes	No	No	Eschar	(*13*)
20	Spain	2011/2013	40/F	Yes	Horseback riding	April	Neck	1	Yes	Yes	No	Eschar	(*13*)
21	Spain	2011/2013	75/F	No	Walking in rural area	July	Leg	1	Yes	Yes	Yes	Eschar	(*13*)
22	France	2009/2010	52/M	No	Trip to Egypt	September	Scapular area	1	Yes	No	No	Eschar	(*14*)
23	Spain	NA/2011	9/M	No	Rural environment	September /October	Scalp	1	Yes	No	No	Eschar	(*15*)
24	Spain	NA/2011	73/M	Yes	Working in vegetable patch	August	Back	1	Yes	Yes	No	Eschar	(*16*)
25	Spain	2011/2012	69/M	No	Vacation in rural area	September	Hip	1	Yes	Yes	No	Blood	(*2*)
26	France	2011/2013	73/M	No	Contact with animals	February	Armpit	1	Yes	Yes	No	NA	(*17*)
27	France	2011/2013	67/F	No	Contact with animals	April	Leg	1	Yes	No	Yes	NA	(*17*)
28	France	2011/2013	NA/M	No	Contact with animals	March	NA	1	Yes	NA	NA	NA	(*17*)
29	France	2012/2014	16/M	No	Fishing	May	Lower eyelid, forearm	2	Yes	No	No	Eschar swab	(*18*)
30–49	France	2010–2014/2016	Median age (±SD) 43 (±21) [IQR 2–70]; F, n = 8; M, n = 12	No, n = 14; Yes, n = 6	Tick bites or handling, n = 6; outdoor activities, n = 5; frequent contact with dogs, n = 8	April–June, n = 11; July–September, n = 8; October, n = 1	Lower limbs, n = 7; upper limbs, n = 4; trunk, n = 3; neck, n = 4	≥1, n = 20; Multiple, n = 3	Yes	Yes, n = 19; no, n = 1	Yes, n = 7; no, n = 13	Eschar swab, n = 13; skin biopsy, n = 10	(*19*)
50	France	2013/2015	61/M	Yes	Insect bite	June	Forearm	1	Yes	Yes	No	Eschar	(*20*)
51	North Greece	2013/2016	13/M	No	Holidays, walking to the woods	May	Upper chest,	1	Yes	Yes	Yes	None‡	(*21*)
**52**	**Spain**	**2014/NP**	**12/M**	**No**	**None**	**May**	**Forearm**	**1**	**Yes**	**Yes**	**Yes**	**Eschar swab**	**NP**
**53**	**Spain**	**2014/NP**	**71/M**	**No**	**None**	**July**	**Groin**	**1**	**Yes**	**No**	**No**	**Eschar swab**	**NP**
54	Spain	NA/2015	46/F	No	Dog with ticks	May	Scalp	1	Yes	Yes	No	Eschar	(*22*)
55	Spain	NA/2015	73/M	No	Rural environment and hunting	July	Abdomen	1	Yes	Yes	No	Eschar	(*23*)
56	Spain	NA/2015	25/M	Yes	Dog with ticks	July	Trunk	1	Yes	No	No	Eschar	(*23*)
57	Spain	2013–2014/2016	6/NA	NA	NA	NA	Scalp	1	Yes	Yes	No	Eschar	(*24*)
58	France, returned from Sri Lanka	2016/2017	30/F	Yes	Jungle	February	Flank	1	Yes	Yes	No	Eschar	(*25*)
59	Spain	2016/2017	39/M	No	Hunting	September	Buttocks	1	Yes	No	No	Eschar, eschar swab	(*3*)

Case no.	Country	Year case occurred/publication	Patient age, y/sex	Tick bite reported	Other epidemiologic condition	Month†	Bite/eschar locations	No. eschars	Fever	Rash	Lymphangitis	PCR+ sample	Ref.
60	Turkey	2016/2017	56/M	Yes, *H. marginatum* female	Farming	May	Umbilicus	1	Yes	Yes	No	Tick	(*26*)
61	France	2016/2018	42/F	No	Gardening	July	Abdominal	2	Yes	No	No	Eschar swab	(*27*)
**62**	**Spain**	**2017/NP**	**76/F**	**No**	**Living with dogs**	**June**	**Scalp**	**2**	**Yes**	**No**	**No**	**Eschar swab, blood**	**NP**
**63**	**Spain**	**2017/NP**	**66/M**	**No**	**Hunting**	**September**	**Foot**	**1**	**Yes**	**No**	**Yes**	**Eschar swab**	**NP**
64	Cameroon	NA/2017	54/F	No	Travel	May	Back	1	Yes	Yes	No	Eschar swab, eschar	(*28*)
**65**	**Spain**	**2018/NP**	**39/M**	**No**	**Rural environment**	**August**	**Forearm**	**1**	**Yes**	**No**	**No**	**Eschar swab**	**NP**
**66**	**Spain**	**2018/NP**	**70/M**	**No**	**None**	**September**	**Shoulder**	**2**	**Yes**	**Yes**	**No**	**Eschar swab, eschar**	**NP**
67	France	2018/2020	66/M	No	Living in rural area with animals	July	Ankle	1	Yes	Yes	Yes	Eschar swab	(*29*)
**68**	**Spain**	**2019/NP**	**50/F**	**No**	**None**	**May**	**Leg/scapula**	**2**	**Yes**	**No**	**No**	**Eschar swab**	**NP**
**69**	**Spain**	**2019/NP**	**61/M**	**No**	**None**	**September**	**Leg**	**2**	**Yes**	**No**	**Yes**	**Eschar swab**	**NP**
**70**	**Spain**	**2020/NP**	**82/M**	**Yes**	**4 ticks attached**	**May**	**Leg**	**2**	**Yes**	**Yes**	**Yes**	**Eschar swab**	**NP**
71	Spain	2019/2020	59/M	No	Rural area and contact with animals	April	Leg	1	Yes	No	Yes	Eschar	(*30*)
72	Spain	2020/2021	11/M	No	Walking with dog	July	Forearm	1	Yes	Yes	Yes	Eschar	(*31*)
73	Spain	2020/2021	5/M	Yes	Rural area and dog with ticks	June	Scrotum	1	Yes	Yes	No	Eschar	(*4*)
**74**	**Spain**	**2020/NP**	**12/F**	**No**	**None**	**August**	**Scalp**	**1**	**Yes**	**Yes**	**Yes**	**Eschar swab**	**NP**
**75**	**Spain**	**2020/NP**	**75/M**	**No**	**None**	**September**	**Ankle**	**1**	**Yes**	**Yes**	**Yes**	**Eschar swab**	**NP**
**76**	**Spain**	**2020/NP**	**34/M**	**No**	**Hunting**	**June**	**Thigh**	**1**	**Yes**	**Yes**	**No**	**Blood**	**NP**
**77**	**Spain**	**2021/NP**	**34/M**	**Yes**	**Tick bite**	**April**	**Thigh/hip**	**1**	**Yes**	**No**	**No**	**Eschar swab**	**NP**
**78**	**Spain**	**2021/NP**	**70/M**	**No**	**None**	**June**	**Buttocks**	**1**	**Yes**	**Yes**	**Yes**	**Eschar**	**NP**
**79**	**Spain**	**2021/NP**	**66/M**	**Yes**	**Tick bite**	**June**	**Unknown**	**None**	**Yes**	**Yes**	**No**	**Blood**	**NP**
**80**	**Spain**	**2021/NP**	**74/M**	**No**	**None**	**June**	**Arm**	**2**	**Yes**	**Yes**	**No**	**Eschar, blood**	**NP**
**81**	**Spain**	**2022/NP**	**65/M**	**No**	**None**	**March**	**Hip**	**1**	**Yes**	**Yes**	**No**	**Eschar, eschar swab**	**NP**

Case no.	Country	Year case occurred/publication	Patient age, y/sex	Tick bite reported	Other epidemiologic condition	Month†	Bite/eschar locations	No. eschars	Fever	Rash	Lymphangitis	PCR+ sample	Ref.
**82**	**Spain**	**2022/NP**	**78/F**	**No**	**Rural environment**	**April**	**Face**	**1**	**Yes**	**No**	**No**	**Eschar swab**	**NP**
**83**	**Spain**	**2022/NP**	**52/M**	**No**	**Cat scratch**	**May**	**Arm**	**1**	**Yes**	**No**	**Yes**	**Eschar swab**	**NP**
84	Spain	NA/2022	4/M	NA	NA	NA	Thigh	1	Yes	No	Yes	Eschar	(*32*)
85	Spain	NA/2022	6/M	No	None	NA	Scalp	2	Yes	No	No	Eschar	(*33*)
86	North Macedonia	2022/2022	60/F	*Hyalomma* sp.	Living in rural area	June	Armpit	1	Yes	Yes	No	Blood	(*34*)
87	Spain	2021/2023	29/M	Insect bite	Holidays in rural area	June	Forearm	1	Yes	No	Yes	Eschar	(*35*)
88	Spain	NA/2023	75/M	No	Living in rural area	NA	Neck	1	Yes	Yes	No	Eschar	(*36*)
**89**	**Spain**	**2023/NP**	**57/M**	**Yes**	**Tick bite**	**May**	**Leg**	**1**	**Yes**	**No**	**Yes**	**Eschar swab**	**NP**
**90**	**Spain**	**2023/NP**	**74/M**	**Yes**	**Tick bite**	**July**	**Abdomen/groin**	**2**	**Yes**	**Yes**	**No**	**Eschar swab**	**NP**
**91**	**Spain**	**2023/NP**	**68/M**	**No**	**Vegetable patch**	**July**	**Iliac fossa**	**1**	**Yes**	**No**	**No**	**Eschar swab, blood**	**NP**
**92**	**Spain**	**2023/NP**	**69/F**	**No**	**Gardening**	**September**	**Abdomen**	**1**	**Yes**	**Yes**	**No**	**Eschar swab**	**NP**
**93**	**Spain**	**2023/NP**	**70/M**	**Yes, *R. pusillus*)**	**Gardening**	**July**	**Armpit**	**1**	**Yes**	**No**	**No**	**Tick attached**	**NP**
**94**	**Spain**	**2024/NP**	**46/F**	**No**	**None**	**April**	**Buttocks**	**1**	**Yes**	**No**	**No**	**Eschar**	**NP**

*Bold text indicates cases not previously published. Data are from patients with *R. sibirica mongolitimonae* infection diagnosed at the Center for Rickettsiosis and Arthropod-Borne Diseases in Rioja, Spain, during 2007–2024 and from literature published in PubMed during 1996–2024. NA, not available; NP, not published; Ref, reference; *R. pusillus*, *Rhipicephalus pusillus tick;* +, positive.
†Month patients were bitten or month of symptom onset.
‡Microbiologic diagnosis by seroconversion.

References

1. Aguirrebengoa K, Portillo A, Santibáñez S, Marín JJ, Montejo M, Oteo JA. Human *Rickettsia sibirica mongolitimonae* infection, Spain. Emerg Infect Dis. 2008;14:528–9. PubMed https://doi.org/10.3201/eid1403.070987

2. Ibarra V, Portillo A, Palomar AM, Sanz MM, Metola L, Blanco JR, et al. Septic shock in a patient infected with *Rickettsia sibirica mongolitimonae*, Spain. Clin Microbiol Infect. 2012;18:E283–5. PubMed https://doi.org/10.1111/j.1469-0691.2012.03887.x

3. Revilla-Martí P, Cecilio-Irazola Á, Gayán-Ordás J, Sanjoaquín-Conde I, Linares-Vicente JA, Oteo JA. Acute myopericarditis associated with tickborne *Rickettsia sibirica mongolitimonae.* Emerg Infect Dis. 2017;23:2091–3. PubMed https://doi.org/10.3201/eid2312.170293

4. Salazar Alarcón E, Guillén-Martín S, Callejas-Caballero I, Valero-Arenas A. Clinical case report: not all rickettsiosis are mediterranean spotted fever. Enferm Infecc Microbiol Clin (Engl Ed). 2022;40:44–5. PubMed https://doi.org/10.1016/j.eimce.2021.10.004

5. Raoult D, Brouqui P, Roux V. A new spotted-fever-group rickettsiosis. Lancet. 1996;348:412. PubMed https://doi.org/10.1016/S0140-6736(05)65037-4

6. Fournier PE, Tissot-Dupont H, Gallais H, Raoult DR. *Rickettsia mongolotimonae*: a rare pathogen in France. Emerg Infect Dis. 2000;6:290–2. PubMed https://doi.org/10.3201/eid0603.000309

7. Pretorius AM, Birtles RJ. *Rickettsia mongolotimonae* infection in South Africa. Emerg Infect Dis. 2004;10:125–6. PubMed https://doi.org/10.3201/eid1001.020662

8. Fournier PE, Gouriet F, Brouqui P, Lucht F, Raoult D. Lymphangitis-associated rickettsiosis, a new rickettsiosis caused by *Rickettsia sibirica mongolotimonae*: seven new cases and review of the literature. Clin Infect Dis. 2005;40:1435–44. PubMed https://doi.org/10.1086/429625

9. Psaroulaki A, Germanakis A, Gikas A, Scoulica E, Tselentis Y. Simultaneous detection of *Rickettsia mongolotimonae* in a patient and in a tick in Greece. J Clin Microbiol. 2005;43:3558–9. PubMed https://doi.org/10.1128/JCM.43.7.3558-3559.2005

10. de Sousa R, Barata C, Vitorino L, Santos-Silva M, Carrapato C, Torgal J, et al. *Rickettsia sibirica* isolation from a patient and detection in ticks, Portugal. Emerg Infect Dis. 2006;12:1103–8. PubMed https://doi.org/10.3201/eid1207.051494

11. Caron J, Rolain JM, Mura F, Guillot B, Raoult D, Bessis D. *Rickettsia sibirica* subsp. *mongolitimonae* infection and retinal vasculitis. Emerg Infect Dis. 2008;14:683–4. PubMed https://doi.org/10.3201/eid1404.070859

12. de Sousa R, Duque L, Anes M, Poças J, Torgal J, Bacellar F, et al. Lymphangitis in a Portuguese patient infected with *Rickettsia sibirica.* Emerg Infect Dis. 2008;14:529–30. PubMed https://doi.org/10.3201/eid1403.070680

13. Ramos JM, Jado I, Padilla S, Masiá M, Anda P, Gutiérrez F. Human infection with *Rickettsia sibirica mongolitimonae*, Spain, 2007–2011. Emerg Infect Dis. 2013;19:267–9. PubMed https://doi.org/10.3201/eid1902.111706

14. Socolovschi C, Barbarot S, Lefebvre M, Parola P, Raoult D. *Rickettsia sibirica mongolitimonae* in traveler from Egypt. Emerg Infect Dis. 2010;16:1495–6. PubMed https://doi.org/10.3201/eid1609.100258

15. Morales V, Garcia Acebes CR, Miguelez Hernandez AP, Alfageme Roldan F, Rodriguez Albarran A, Alins Sahun Y, et al. Infección por *Rickettsia sibirica* subsp. *monogolitimonae*. [*Rickettsia sibirica* subsp. *mongolitimonae* infection]. Piel. Formación continuada en dermatología. 2011;26:224–6. https://doi.org/10.1016/j.piel.2011.01.005

16. Fleta-Asín B, Alonso-Castro L, Jado-García I, Anda-Fernández P. Detection by polymerase chain reaction of *Rickettsia sibirica mongolotimonae* in the skin biopsy of a rash: a case report [in Spanish]. Enferm Infecc Microbiol Clin. 2011;29:778–9. PubMed https://doi.org/10.1016/j.eimc.2011.05.011

17. Edouard S, Parola P, Socolovschi C, Davoust B, La Scola B, Raoult D. Clustered cases of *Rickettsia sibirica mongolitimonae* infection, France. Emerg Infect Dis. 2013;19:337–8. PubMed https://doi.org/10.3201/eid1902.120863

18. Solary J, Socolovschi C, Aubry C, Brouqui P, Raoult D, Parola P. Detection of *Rickettsia sibirica mongolitimonae* by using cutaneous swab samples and quantitative PCR. Emerg Infect Dis. 2014;20:716–8. PubMed https://doi.org/10.3201/eid2004.130575

19. Angelakis E, Richet H, Raoult D. *Rickettsia sibirica mongolitimonae* infection, France, 2010–2014. Emerg Infect Dis. 2016;22:880–2. PubMed https://doi.org/10.3201/eid2205.141989

20. Gaillard E, Socolovschi C, Fourcade C, Lavigne JP, Raoult D, Sotto A. A case of severe sepsis with disseminated intravascular coagulation during Rickettsia sibirica mongolitimonae infection [in French]. Med Mal Infect. 2015;45:57–9. PubMed https://doi.org/10.1016/j.medmal.2014.10.005

21. Chochlakis D, Mantadakis E, Thomaidis S, Tselenti Y, Chatzimichael A, Psaroulaki A. First human case of *Rickettsia sibirica mongolotimonae* infection in northern Greece. Isr Med Assoc J. 2016;18:544–6. PubMed

22. Nogueras MM, Roson B, Lario S, Sanfeliu I, Pons I, Anton E, et al. Coinfection with *Rickettsia sibirica* subsp. *mongolotimonae* and *Rickettsia conorii* in a human patient: a challenge for molecular diagnosis tools. J Clin Microbiol. 2015;53:3057–62. PubMed https://doi.org/10.1128/JCM.00457-15

23. Pulido-Pérez A, Gómez-Recuero L, Lozano-Masdemont B, Suárez-Fernández R. *Rickettsia sibirica mongolitimonae* infection in two immunocompetent adults [in Spanish]. Enferm Infecc Microbiol Clin. 2015;33:635–6. PubMed https://doi.org/10.1016/j.eimc.2015.03.006

24. Monterde-Álvarez ML, Calbet-Ferré C, Rius-Gordillo N, Pujol-Bajador I, Ballester-Bastardie F, Escribano-Subías J. Rickettsiosis after tick bite: a subtle clinic picture on many occasions, we must be vigilant [in Spanish]. Enferm Infecc Microbiol Clin. 2017;35:100–3. PubMed https://doi.org/10.1016/j.eimc.2016.01.013

25. Cordier C, Tattevin P, Leyer C, Cailleaux M, Raoult D, Angelakis E. *Rickettsia sibirica mongolitimonae* infection, Sri Lanka. J Infect Dev Ctries. 2017;11:668–71. PubMed https://doi.org/10.3855/jidc.8743

26. Kuscu F, Orkun O, Ulu A, Kurtaran B, Komur S, Inal AS, et al. *Rickettsia sibirica mongolitimonae* infection, Turkey, 2016. Emerg Infect Dis. 2017;23:1214–6. PubMed https://doi.org/10.3201/eid2307.170188

27. Rajoelison P, Mediannikov O, Javelle E, Raoult D, Parola P, Aoun O. *Rickettsia sibirica mongolitimonae* human infection: a diagnostic challenge. Travel Med Infect Dis. 2018;26:72–3. PubMed https://doi.org/10.1016/j.tmaid.2018.07.002

28. Nouchi A, Monsel G, Jaspard M, Jannic A, Angelakis E, Caumes E. *Rickettsia sibirica mongolitimonae* infection in a woman travelling from Cameroon: a case report and review of the literature. J Travel Med. 2018;25. PubMed https://doi.org/10.1093/jtm/tax074

29. Loarte MDC, Melenotte C, Cassir N, Cammilleri S, Dory-Lautrec P, Raoult D, et al. *Rickettsia mongolitimonae* encephalitis, southern France, 2018. Emerg Infect Dis. 2020;26:362–4. PubMed https://doi.org/10.3201/eid2602.181667

30. Miguélez Ferreiro S, Navalpotro Rodríguez D. Tubular acute lymphangitis caused by *Rickettsia sibirica mongolitimonae.* Enferm Infecc Microbiol Clin (Engl Ed). 2020;38:506–7. PubMed https://doi.org/10.1016/j.eimce.2020.10.001

31. Echevarría-Zubero R, Porras-López E, Campelo-Gutiérrez C, Rivas-Crespo JC, Lucas AM, Cobo-Vázquez E. Lymphangitis-associated rickettsiosis by *Rickettsia sibirica mongolitimonae.* J Pediatric Infect Dis Soc. 2021;10:797–9. PubMed https://doi.org/10.1093/jpids/piab018

32. Vázquez-Pérez Á, Rodríguez-Granger J, Calatrava-Hernández E, Santos-Pérez JL. Pediatric tubular acute lymphangitis caused by *Rickettsia sibirica mongolitimonae*: case report and literature review. Enferm Infecc Microbiol Clin (Engl Ed). 2022;40:218–9. PubMed https://doi.org/10.1016/j.eimce.2021.10.007

33. Cemeli Cano M, Rodríguez Sanz L, Vidal Lana P, Arlabán Carpintero L, Laliena Aznar S, Jiménez Pérez E. Fiebre botonosa mediterránea “like” por *Rickettsia sibirica mongolitimonae* en la consulta de atención primaria. Rev Latin Infect Pediatr. 2022;35:122–4. https://doi.org/10.35366/108136

34. Jakimovski D, Mateska S, Simin V, Bogdan I, Mijatović D, Estrada-Peña A, et al. Mediterranean spotted fever-like illness caused by *Rickettsia sibirica mongolitimonae*, North Macedonia, June 2022. Euro Surveill. 2022;27:2200735. PubMed https://doi.org/10.2807/1560-7917.ES.2022.27.42.2200735

35. Davila-Arias S, Rabadan-Rubio E, Rabadan-Rubio A, Arranz-Caso JA. *Rickettsia sibirica mongolotimonae* infection in Cantabria, Spain. Enferm Infecc Microbiol Clin (Engl Ed). 2023;41:520–1. PubMed https://doi.org/10.1016/j.eimce.2023.04.001

36. Lasierra Lavilla I, Caballero Castro JP, Zabala López SI. *Rickettsia sibirica* infection. Med Clin (Barc). 2023;160:460–1. PubMed https://doi.org/10.1016/j.medcli.2022.12.012
